# Overlapping tumor‐specific expression of p53, p16^INK4a^, and sirtuin 1 in Bowen's disease: A case report

**DOI:** 10.1002/ccr3.3400

**Published:** 2020-10-27

**Authors:** Tomoaki Takada

**Affiliations:** ^1^ Sumikawa Takada Dermatology Clinic Sapporo‐shi Japan

**Keywords:** Bowen's disease, immunohistochemical stain, p16^INK4a^, p53, sirtuin 1/SIRT1

## Abstract

Understanding the impact of inactivating mutations in *SIRT1* on the *p53* and *p16* tumor suppressor genes may yield new insight into the oncogenic mechanisms underlying Bowen's disease.

## Graphical abstract

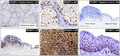

## 1 INTRODUCTION

The tumor of a patient with Bowen's disease exhibited expression of the tumor suppressors p53 and p16^INK4a^ (p16), which is correlated with cytoplasmic expression of the histone deacetylase sirtuin 1 (SIRT1). Epigenetic regulation of p53 and p16 by SIRT1 may play a role in the carcinogenesis of Bowen's disease.

Sirtuin 1 (SIRT1), a nicotinamide adenine dinucleotide–dependent class‐III histone deacetylase, is a multifunctional protein that plays various roles in the tumorigenesis of many cancer types. *SIRT1* plays dual roles as both a tumor promoter and suppressor depending on the regulation of its target genes *p53* and *p16*.^1^, ^2^, ^3^ Indeed, p53 and p16 play important roles in the carcinogenesis of Bowen's disease (BD), an intraepidermal form of cutaneous squamous cell carcinoma (SCC).^4^, ^5^ *SIRT1* overexpression may block senescence by direct deacetylation of p53 and p16 and consequent activation of the Akt/p70S6K1 signaling pathway, which in turn decreases *p16* expression levels.^2^, ^3^ The spectrum of *p53* tumor suppressor gene mutations found in BD in Japanese and Caucasian populations is contrasting in nature, matching the different etiologies of BD in the respective populations.

In general, people of Japanese ethnicity have a much lower incidence of nonmelanoma skin cancer (NMSC) than Caucasians due to relative protection from ultraviolet radiation (UVR) conferred by differences in skin types. However, the unexpectedly high prevalence of frameshift mutations in Japanese patients with BD suggests that environmental mutagens other than UVR that preferentially induce deletion or insertion mutations may play an important role in the tumorigenesis of Japanese BD.^4^

Sirtuin 1 overexpression provides a cell survival advantage by inhibiting apoptosis. SIRT1‐mediated deacetylation of lysine residue 382 of the p53 tumor suppressor protein limits the transactivation function of p53, thereby impeding the ability of p53 to induce apoptosis. In addition to p53, SIRT1 also deacetylates and represses the activity of forkhead transcription factors and consequently reduces forkhead‐dependent apoptosis.^6^, ^7^, ^8^

The *p16* tumor suppressor gene and *cyclin A*, which are repressed by the retinoblastoma tumor suppressor gene (*RB1*) pathway, are frequently overexpressed in BD compared with actinic keratosis. This is likely due to frequent dysregulation of the RB1 pathway in BD, which transcriptionally upregulates the expression of cyclin A through the E2F1 transcription factor via RB1 inactivation. Since cyclin A is involved in this initiation of DNA replication and is required for G2‐M transition during cell cycle progression, overexpression of cyclin A contributes to the high proliferative activity in tumor cells of BD.

The expression of *SIRT1* is also transcriptionally induced via E2F1‐mediated inactivation of RB1, and SIRT1 inhibits the apoptotic function of E2F1 through a negative feedback loop. Thus, strong expression of SIRT1 is associated with dysregulation of the RB1 pathway and likely contributes to the pathogenesis of BD by promoting cellular growth and inhibiting apoptosis.^9^, ^10^, ^11^, ^12^ However, the roles of SIRT1 in BD carcinogenesis have not been investigated.

To date, overexpression of SIRT1 in BD has only been assessed based on the abundance of nuclear SIRT1.^13^ However, to the best of our knowledge, a detailed analysis of the subcellular localization of SIRT1 and its correlations with p53 and p16 expression have not been reported. The dual nature of SIRT1 in cancer remains a matter of debate and could be a consequence of several factors, including differential expression of SIRT1 in various types of carcinoma, variations in SIRT1 subcellular location, and diverse downstream substrates of SIRT1. Earlier studies predominately reported nuclear SIRT1 expression levels, whereas more recent studies have included assessments of cytoplasmic SIRT1 expression levels. Thus, it has been proposed that subcellular localization may account for the dual roles of SIRT1 in normal vs cancer cells^14^; however, the cytoplasmic overexpression of SIRT1 in BD has not yet been reported in the literature. Therefore, we analyzed the expression patterns of SIRT1, p53, and p16 in a tumor from a patient with BD and adjacent normal tissue using immunohistochemistry (IHC).

## 2 CASE PRESENTATION

### 2.1 Case history and examination

**FIGURE 1:**
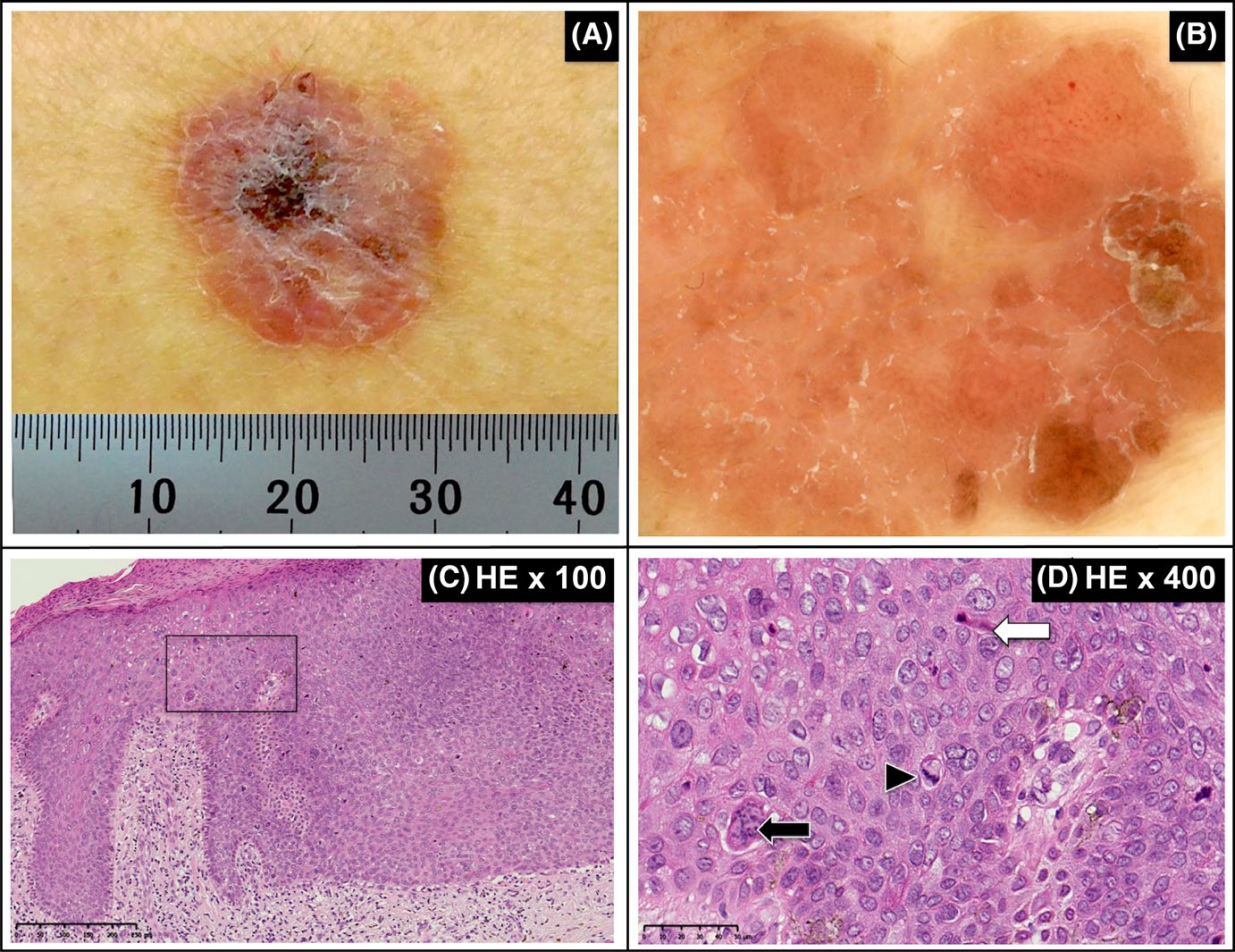
Clinical, dermoscopic, and histopathological findings. A, The clinical presentation was an erythematous scaly patch. B, Glomerular vessels and scales were recognized by dermoscopy. C, Dense growth of atypical keratinocytes was observed in all layers (H&E, 100× original magnification, scale bar: 250 μm). D, Clumping cells (closed arrow), dyskeratotic cells (open arrow), and mitotic figures (closed triangle) were observed (H&E, 400× magnification, scale bar: 50 μm)

The eruption was recognized in the trunk of a 77‐year‐old woman, which she first noticed more than 10 years ago. It started to increase in size 4 months prior to her visit to the hospital. Clinical imaging showed the presence of erythematous plaques with scales (Figure 1A). Dermoscopy (DZ‐100; Yamagata Casio Co., Ltd.) showed the presence of glomerular vessels, milky‐red areas, diffuse depigmentation, and scaling (Figure 1B).

### 2.2 Differential diagnosis, investigation, and treatment

A clinical diagnosis of BD was made, and surgical resection of the affected tissue along with a 2‐mm margin of normal tissue was performed. Written informed consent was obtained from the patient for the use of the tissue samples in this study. Histopathological examination of the affected skin specimen using hematoxylin and eosin (H&E) staining revealed dense growth of atypical keratinocytes in all layers (Figure 1C). Multinucleated cells, abnormal keratinocytes, and mitotic figures were also observed (Figure 1D), and the final pathology was diagnosed as BD. Virological examination revealed that the sample was negative for human papillomavirus and Epstein‐Barr virus based on PapiPlex testing and viral small‐RNA in situ hybridization, respectively.

Immunohistochemistry was performed with Anti‐Human Sir2/SIRT1 Rabbit IgG Affinity Purify (18761; Immuno‐Biological Laboratories Co., Ltd.), Monoclonal Mouse Anti‐Human p53 Protein (IR616; Agilent), Monoclonal Mouse Anti‐Human p16INK4 (932‐540M‐EN; BioGenex Laboratories), and Monoclonal Mouse Anti‐Human Ki‐67 Antigen (IR626; Agilent) antibodies. Resected sebaceous tissue expressing SIRT1 was used as a positive control, while Rabbit IgG, polyclonal‐Isotype Control (ab37415; Abcam) was used as a negative control. Staining evaluation followed the standard scores of the World Health Organization: <20% (nucleus) or <25% (cytoplasm) stained cells indicated negative/low expression; 20%‐50% (nucleus) or 25%‐50% (cytoplasm) indicated moderate expression; and >50% (nucleus, cytoplasm) indicated high expression.

**FIGURE 2:**
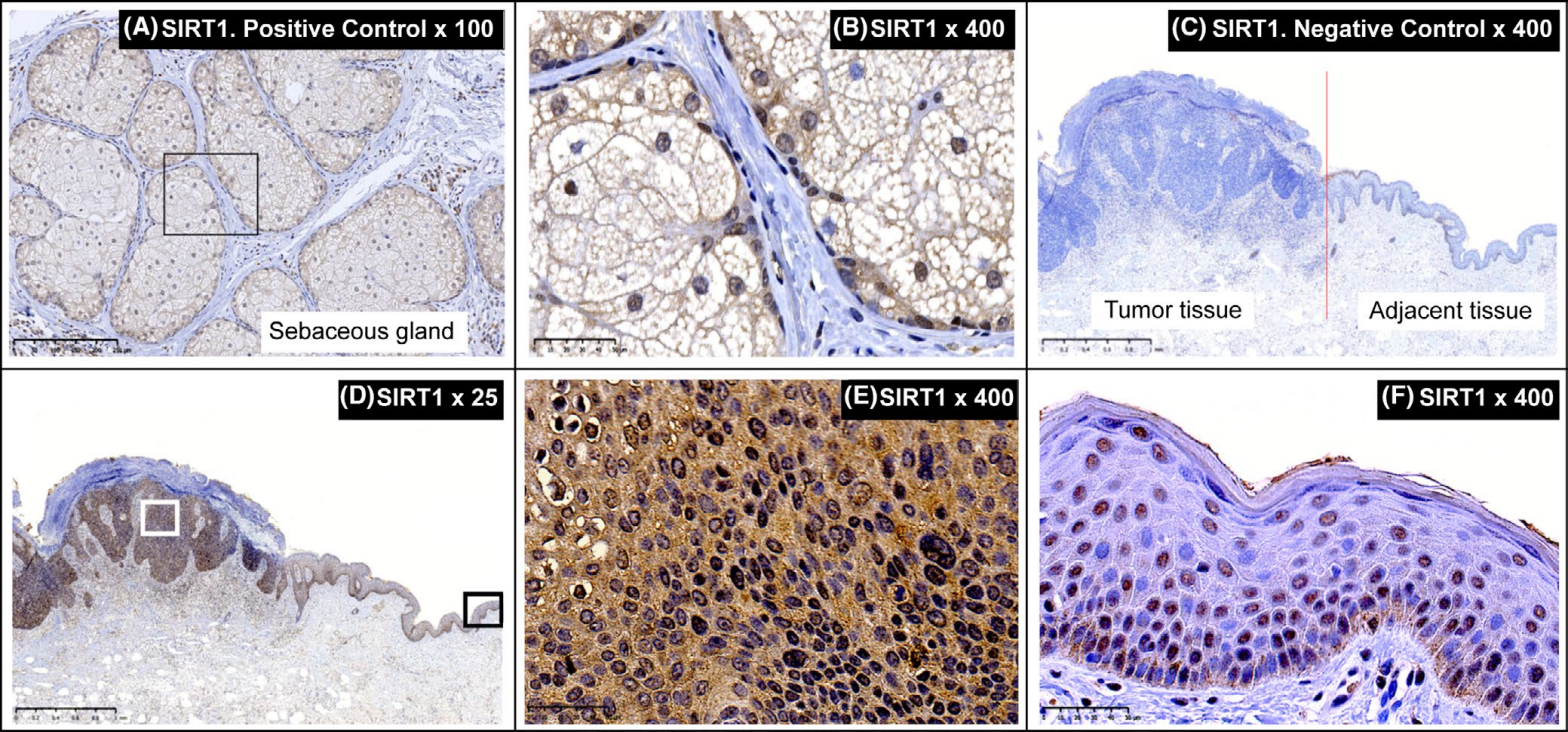
Immunohistochemical staining of SIRT1. A, B, Sebaceous gland tissues were used as positive controls. A, SIRT1 stain, 100× magnification, scale bar: 250 μm. B, SIRT1 stain, 400× magnification, scale bar: 50 μm. C, Negative control, tumor tissues on the left and adjacent normal tissues on the right; red vertical line indicates the boundary (rabbit IgG, polyclonal‐isotype control, 25× magnification, scale bar: 1 mm). D, Tumor tissues on the left and adjacent normal tissues on the right (SIRT1 stain, 25× magnification, scale bar: 1 mm). E, Tumor tissues (SIRT1 stain, 400× magnification, scale bar: 50 μm). F, Adjacent tissues (SIRT1 stain, 400× magnification, scale bar: 50 μm)

**FIGURE 3:**
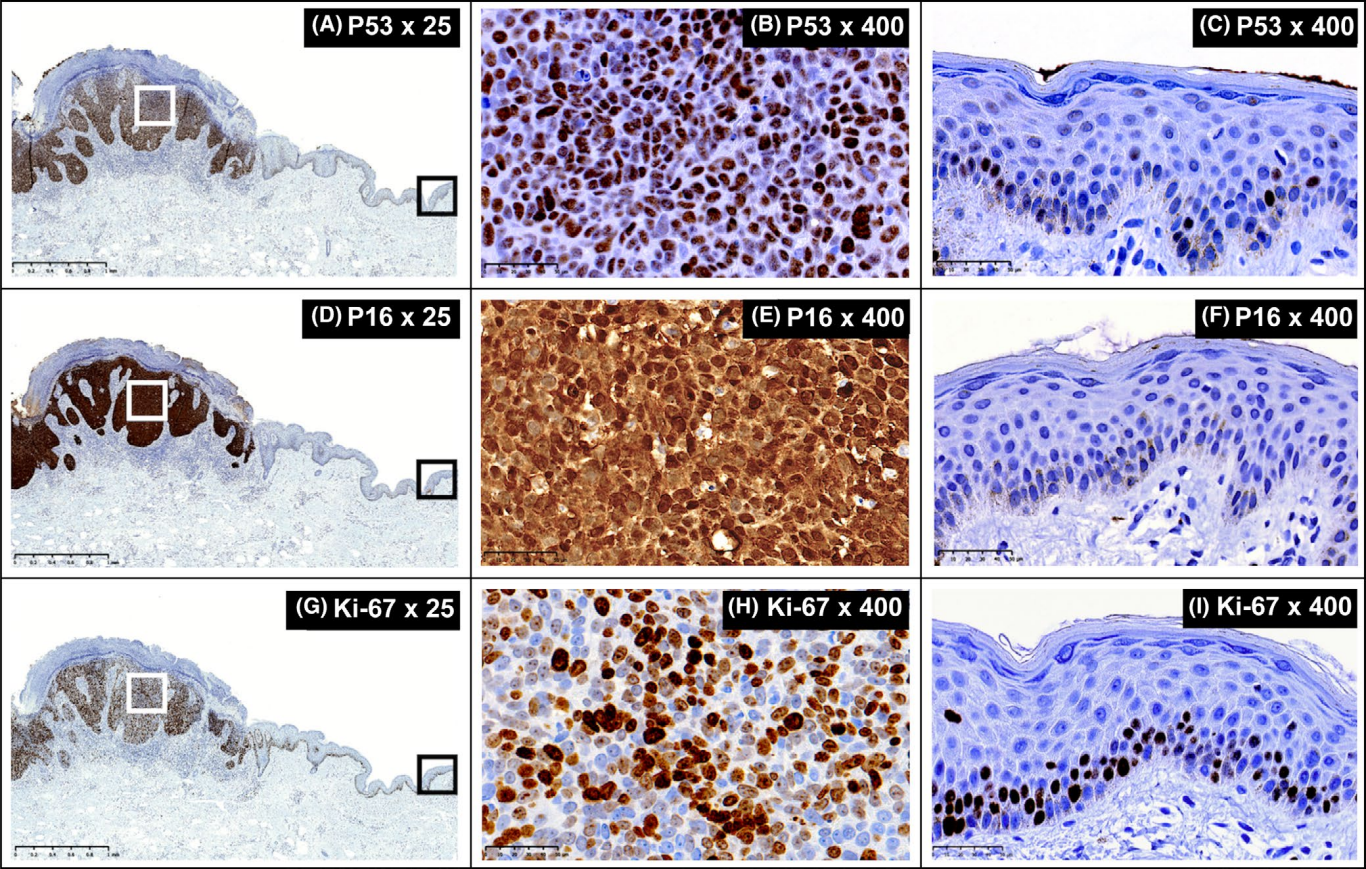
Immunohistochemical staining of A–C, p53; D–F, p16^INK4a^; and G–I, Ki‐67. A, Tumor tissues on the left and adjacent normal tissues on the right (p53 stain, 25× magnification, scale bar: 1 mm). B, Tumor tissues (p53 stain, 400× magnification, scale bar: 50 μm). C, Adjacent normal tissues (p53 stain, 400× magnification, scale bar: 50 μm). D, Tumor tissues on the left and adjacent normal tissues on the right (p16 stain, 25× magnification, scale bar: 1 mm). E, Tumor tissues (p16 stain, 400× magnification, scale bar: 50 μm). F, Adjacent tissues (p16 stain, 400× magnification, scale bar: 50 μm). G, Tumor tissues on the left and adjacent normal tissues on the right (Ki‐67 stain, 25× magnification, scale bar: 1 mm). H, Tumor tissues (Ki‐67 stain, 400× magnification, scale bar: 50 μm). I, Adjacent tissues (Ki‐67 stain, 400× magnification, scale bar: 50 μm)

In the positive control sample, SIRT1 expression was moderate in the nucleus and negative in the cytoplasm (Figure 2A, B). The negative control showed neither nuclear nor cytoplasmic expression in the tumor tissue or adjacent normal skin tissue (Figure 2C). The patient's tumor tissue showed moderate nuclear and high cytoplasmic SIRT1 expression (Figure 2D, E), while the adjacent normal tissue showed moderate nuclear SIRT1 expression and no cytoplasmic expression (Figure 2D, F). Staining for p53 (Figure 3A‐C) and Ki‐67 (Figure 3G‐I) showed high expression of both proteins in tumor‐associated nuclei and low expression in the nuclei of cells along the epidermal basal layer in the adjacent normal skin tissue; there was no cytoplasmic expression of either protein in either cell type. Staining for p16 (Figure 3D‐F) showed high expression in both the nuclei and the cytoplasm of tumor tissues, but no expression in the adjacent normal skin tissue.

### 2.3 Outcome and follow‐up

Histopathologically, the final diagnosis was BD, which was completely curatively resected. The postoperative course was encouraging, showing no evidence of a local recurrence. At present, 6 months after the operation, no findings suggesting a recurrence have been recognized and careful postoperative follow‐up will be planned in the future. To avoid recurrence in the future, oral nicotinamide (NAM) or local therapy, without invasion such as palliative surgery, is planned.

## 3 DISCUSSION

Basal cell carcinoma and SCC are well‐known nonmelanoma skin cancers. It is estimated that more than 5 million nonmelanoma skin cancer cases were treated in the United States in 2012 alone, approximately half of which were cutaneous SCC (cSCC). SCC is the second most common form of skin cancer, and the incidence of cSCC has risen more rapidly than that of basal cell carcinoma, closing the incidence gap between the two diseases.^15^ SCC is the next most common cutaneous malignancy after basal cell carcinoma in Japan, and its incidence has been increasing in accordance with the aging population. Although there are no clear statistics on its incidence in Japan, SCC occurs in approximately 2.5 cases per 100 000 each year, which is approximately 1.5 to 2 times the incidence of malignant melanoma.^16^ The primary goals of cSCC treatment are complete removal of the tumor and maximal preservation of function and cosmesis.

Bowen's disease involves the occurrence of cSCC in situ, whereas actinic keratosis is characterized by precancerous lesions induced by sunlight/ultraviolet light exposure. In either case, lesions that are left untreated can progress to invasive cSCC with the potential for metastasis. The primary treatment for BD is surgical resection; other alternative therapies, including topical fluorouracil, topical imiquimod, photodynamic therapy, and cryotherapy, are recommended but may have lower cure rates.^15^, ^16^ In Japan, evidence supporting an optimal degree of resection is limited and a resection margin ranging between 1 and 4 mm is recommended; however, this requires future investigations because there are no systematic reviews or randomized controlled trials evaluating the extent of resection that is optimal for the treatment of BD. In addition, the extent of resection recommended for BD is not specified in any guideline.^16^ In the present case, resection included a 2‐mm margin of normal tissue measured macroscopically. Future follow‐up, including paying attention to local recurrence, will be necessary.

Skin tumors are thought to arise through the accumulation of genetic and epigenetic events in normal skin cells.^17^ The inactivation of p53 in epidermal keratinocytes is considered to be one of the earliest events in cSCC tumorigenesis, and the epidermal keratinocytes that lead to tumorigenesis seem to be stem cells.^18^, ^19^ In the present case, the normal skin tissue adjacent to the tumor tissue—that was likely to have become neoplastic if left untreated due to an increase in tumor size over the past 4 months—was negative for nuclear and cytoplasmic p16 expression, but the nuclei of cells along the basal cell layer stained positive for p53 and Ki‐67 at a 2‐mm resection margin. These cells also showed moderate SIRT1 expression, which is an early indicator of carcinogenesis in BD.

Nicotinamide has been suggested to be effective in reducing or preventing the development of SCCs.^15^, ^20^ NAM has been shown to enhance DNA repair after UVR, which may occur through the regulation of tumor suppressor genes such as *p53* and regulation of SIRT1.^21^ Complete histologic resection is defined as the absence of cancer cells at the resected margin as was observed in this case, but this does not imply that recurrence can be ruled out.^16^ The local recurrence rate of BD was reported to be 4.5%‐19% in one study.^22^

In the tumor tissue, SIRT1 showed strong cytoplasmic expression and only moderate nuclear expression. This distribution coincided nearly perfectly with the staining for p16. While p16 was strongly expressed in both the cell nucleus and the cytoplasm, p53 and Ki‐67 showed strong nuclear expression. In contrast, the normal tissue showed no cytoplasmic SIRT1 expression and only moderate nuclear expression; almost no p53, p16, or Ki‐67 expression was detected.

The immunostaining patterns of the cancer‐associated proteins p53 and p16 along with the proliferative marker Ki‐67 in the present BD case may indicate the presence of inactivating mutations in these tumor suppressor genes, leading to tumorigenic roles. SIRT1 was mainly localized in the cytoplasm of cancerous cells and in the nuclei of normal cells. Thus, SIRT1 overexpression in cancerous cells was mainly due to elevated cytoplasmic SIRT1. Aberrant cytoplasmic localization is a cancer‐specific alteration of SIRT1, and recent studies have suggested that cancer‐specific SIRT1 targets cytoplasmic proteins.^23^ Previous studies have associated p53 and p16 overexpression with the carcinogenesis of BD,^4^, ^5^ and SIRT1 is more strongly expressed in BD than in nonmalignant melanoma skin cancers.^13^ However, to the best of our knowledge, this is the first study to investigate the expression patterns of p53, p16, and SIRT1 by IHC within the same BD tissue specimen. Yet, a major limitation of this study is that the data are from only one patient and may not be generalizable to a larger cohort; additionally, The Cancer Genome Atlas analyses are required to support these findings.

Although the nucleocytoplasmic shuttling of SIRT1 was discovered more than a decade ago, the role of subcellular SIRT1 localization in tumor progression remains unclear. Shuttling of SIRT1 between the nucleus and cytoplasm is dependent on two nuclear localization signals and two nuclear export signals. Since SIRT1 can play different roles depending on its subcellular localization, which also varies by tissue, it is plausible that SIRT1 exerts diverse effects in different types of tumors. The role of SIRT1 in the promotion of DNA repair and maintenance of genomic stability is clear, but its relationship with the induction of tumorigenesis is not fully understood. Studies that elucidate these roles of SIRT1 are predicted to provide a breakthrough in cancer biology.^24^, ^25^, ^26^

Recently, Bajpai et al reported an extremely rare case of intraosseous basaloid SCC in the mandible.^27^ In their research, they discussed its immunohistochemical profile also using Ber‐Ep4, P63, and EMA. Since basaloid SCC is an aggressive histological subtype of SCC, this study may be beneficial. The expression of N‐cadherin may also be used as a potential biomarker for the early diagnosis of oral SCC.^28^

## 4 CONCLUSION

Tumor tissue from a patient with BD exhibited strong cytoplasmic SIRT1 expression and strong nuclear p53 and p16 expression. Moreover, p16 showed strong cytoplasmic expression in the tumor tissue, but not in the adjacent normal tissue. In normal skin tissue, SIRT1 was moderately expressed only in nuclei, and p53 was expressed only in nuclei along the epidermal basal layers, where the stem cells reside. Overexpression of cytoplasmic SIRT1 correlated with expression of the p53 and p16 tumor suppressors, suggesting that SIRT1 may play an epigenetic role in regulating these genes in BD tumorigenesis. Elucidating the mechanistic actions of epigenetic abnormalities induced by SIRT1 may reveal avenues for therapeutic strategies that target the cancer epigenome and provide more effective adjuvant treatments alongside surgical interventions. This study is a single case report, and the results from our analyses will need to be verified further in an expanded study of multiple cases. Moreover, although the present study focused only on changes in DNA repair mechanisms due to altered nuclear and cytoplasmic SIRT1 localization, not on other factors such as UVR or viruses, it highlights the importance of SIRT1 as a focal point of research in the development of new BD treatments.

## CONFLICT OF INTEREST

None declared.

## AUTHOR CONTRIBUTIONS

The author: wrote the manuscript.
